# Scutellarin: pharmacological effects and therapeutic mechanisms in chronic diseases

**DOI:** 10.3389/fphar.2024.1470879

**Published:** 2024-11-07

**Authors:** Shanshan Nie, Shan Zhang, Ruipeng Wu, Yuhang Zhao, Yongxia Wang, Xinlu Wang, Mingjun Zhu, Peng Huang

**Affiliations:** ^1^ Department of Cardiovascular Disease, The First Affiliated Hospital of Henan University of Chinese Medicine, Zhengzhou, Henan, China; ^2^ Department of Digestive Diseases, The First Affiliated Hospital of Henan University of Chinese Medicine, Zhengzhou, China; ^3^ Department of Integrative Medicine, Huashan Hospital, Fudan University, Shanghai, China; ^4^ Department of Traditional Chinese Medicine, The Seventh Affiliated Hospital, Sun Yat-sen University, Shenzhen, China

**Keywords:** Scutellarin, pharmacological effects, therapeutic potential, chronic diseases, mechanisms

## Abstract

Scutellarin (SCU), a flavonoid glucuronide derived from *Scutellaria barbata* and *Erigeron breviscapus*, exhibits broad pharmacological effects with promising therapeutic potential in treating various chronic diseases. It has demonstrated efficacy in modulating multiple biological pathways, including antioxidant, anti-inflammatory, anti-apoptotic, and vasodilatory mechanisms. These protective roles make SCU a valuable compound in treating chronic diseases such as cerebrovascular diseases, cardiovascular diseases, neurodegenerative disorders, and metabolic diseases. Despite its multi-targeted effects, SCU faces challenges such as low bioavailability and limited clinical data, which hinder its widespread therapeutic application. Current research supports its potential to prevent oxidative stress, reduce inflammatory responses, and enhance cell survival in cells and rats. However, more comprehensive studies are required to clarify its molecular mechanisms and to develop strategies that enhance its bioavailability for clinical use. SCU could emerge as a potent therapeutic agent for the treatment of chronic diseases with complex pathophysiological mechanisms. This review examines the current literature on Scutellarin to provide a comprehensive understanding of its pharmacological activity, mechanisms of action, and therapeutic potential in treating chronic diseases.

## 1 Introduction

Chronic diseases are the major cause of premature adult deaths globally, and older adults are more susceptible to most chronic diseases than younger adults (Su et al., 2023). According to the World Health Organization’s global report, 80% of chronic disease deaths occur in low- and middle-income countries. Approximately one in five people in China was older than 60 in 2020. Currently, Long-term pharmacotherapy is used increasingly to control symptoms and slow disease progression. However, the drugs used in the prevention and treatment have clear targets and certain efficacy, Long-term pharmacotherapy carries the risk of adverse drug reactions that account for more than 5% of acute admissions (Huang and Grady, 2022). Therefore, it is crucial to investigate more potent and safer pharmaceuticals for managing chronic diseases.

**FIGURE 1:**
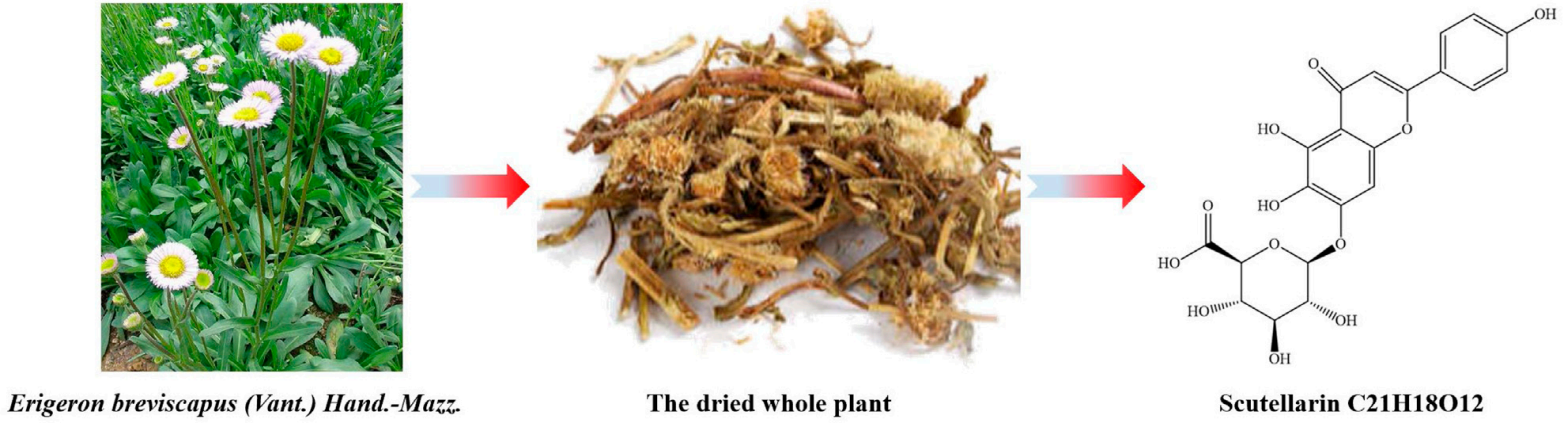
SCU in the dried whole plant of *Erigeron breviscapus*.

*Erigeron breviscapus* (Vant.) Hand.-Mazz. has been used by the Yi minority in Southwest China for treating stroke-induced paralysis and rheumatic joint pain. More recently, modern extraction techniques, such as gas chromatography (GC) and high-performance liquid chromatography (HPLC), have been employed to isolate active components like Scutellarin (SCU) from *E. breviscapus* (Fan et al., 2021). Moreover, it is a flavonoid glycoside compound, named by IUPAC as (2S,3S,4S,5R, 6S)-6-[5,6-dihydroxy-2-(4-hydroxyphenyl)-4-oxochromen-7-yl]oxy-3,4,5- trihy droxyoxane-2-carboxylic acid, with molecular formula C_21_H_18_O_12_ and the molecular weight 462.4 g/mol (Figure 1). Due to its low toxicity and wide availability, SCU exerts a range of diverse pharmacological activities in previous studies, including anti-inflammatory, anti-tumor, anti-apoptotic, anti-oxidation stress, and vasodilatory activities (Ma et al., 2024; Xie et al., 2023; Yuan et al., 2024), making it a promising compound in treating chronic diseases such as cerebrovascular diseases, cardiovascular diseases, neurodegenerative disorders, and metabolic diseases by regulating multiple biological pathways.

However, while the therapeutic potential of SCU is evident, there are still significant barriers to its widespread clinical use. Low bioavailability limits its efficacy when administered orally. Additionally, the current research of SCU remains limited in terms of clinical trials, making it difficult to fully understand its therapeutic mechanisms and long-term effects in humans. More comprehensive studies are required to enhance its bioavailability and clarify its molecular mechanisms in various chronic diseases.

The research on SCU in the past 3 decades has led to the accumulated evidence that establishes its effectiveness in treating chronic diseases. This review aims to provide a comprehensive overview of the pharmacological effects, mechanisms of action, and therapeutic potential of SCU, with a focus on its role in treating chronic diseases. By examining the current literature, we highlight the promise of SCU as a multi-target therapeutic agent and identify the challenges that must be addressed to facilitate its clinical application. This review will serve as a resource for future investigations and facilitate the development of SCU as a therapeutic agent for chronic disease.

## 2 Pharmacological effect and potential mechanism of SCU

Over the past 3 decades, a large-scale effort was made to investigate the pharmacological effects of SCU. A comprehensive literature search was conducted using several databases, including Google Scholar (https://scholar.google.com), Web of Knowledge (https://www.webofknowledge.com), NCBI (https://www.ncbi.nlm.nih.gov), Springer Online Journals (https://link.springer.com), Elsevier Science Direct, and CNKI (https://www.cnki.net), covering publications up to 30 June 2024. Titles, abstracts, and full-text articles were screened to determine their relevance. After removing duplicates and excluding non-relevant articles, the remaining studies were synthesized to form the basis of this review.

**FIGURE 2:**
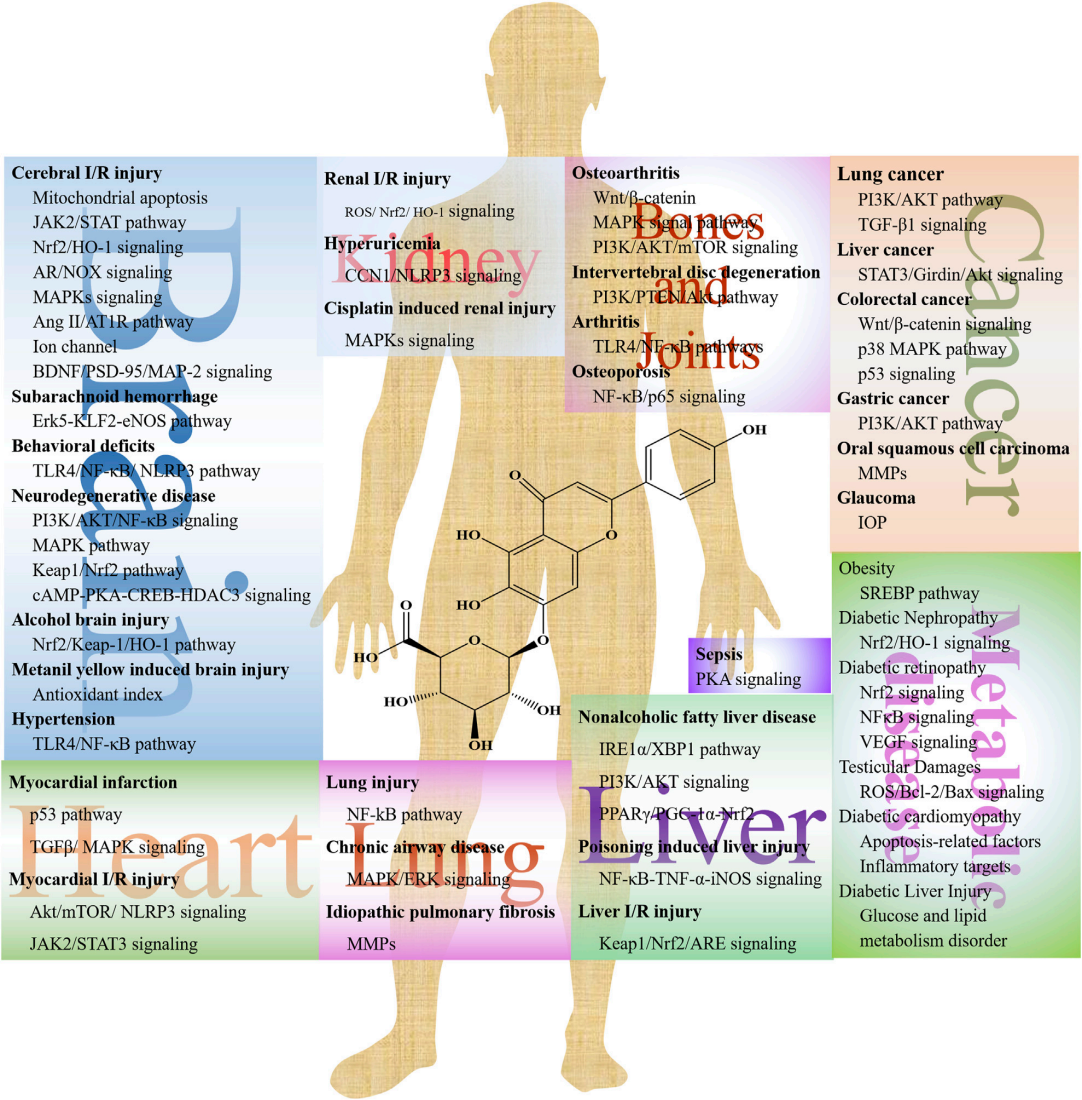
The potential mechanisms of SCU in different diseases.

Numerous studies present the efficacy of SCU on cerebrovascular disease, cardiovascular disease, lung injury, and kidney injury (Figure 2). SCU has been reported to have a broad range of pharmacological effects, including vasodilation, anti-thrombotic action, anti-inflammatory, scavenging of free radicals, and improvement in microcirculation through *in vivo* and *in vitro* experiments. However, its underlying mechanism is still unclear. Table 1 shows the efficacy of SCU on different models, main targets, and diseases.

**TABLE 1:** Summary of preclinical studies evaluating the effects of SCU in different diseases.

Model			Main Target	Disease	Tissue	Reference
Male SD rats	MCAO-induced brain damage	*In vivo*	PARP↓, NAD↓	I/R injury	Brain	Zhang et al. (2009)
Female SD rats	—	*In vivo*	GAP43↑, PTN↓, JAK2↓, STAT↓	I/R injury	Brain	Niu et al. (2021)
Primary cortical neurons		*In vitro*				
SD rats	MCAO-induced brain damage	*In vivo*	p65↓, p38↓, ROS↓, MDA↓, SOD↑, CAT↑	I/R injury	Brain	Zhang et al. (2022f)
			GSH-Px↑, GSH↑, IL-1↓, IL-6↓, TNF-α↓			
Rats	MCAO-induced brain damage	*In vivo*	SOD↑, CAT↑, GSH↑, ROS↓	I/R injury	Brain	Guo et al. (2011a)
Rat cortical neurons		*In vitro*				
C57BL/6N mice	MCAO-induced brain damage	*In vivo*	NOX1↓, NOX2↓, NOX4↓	I/R injury	Brain	Deng et al. (2022)
AR^−/−^ mice						
Male SD rats	MCAO-induced brain damage	*In vivo*	eNOS↑, VEGF↓, bFGF↓, iNOS↓	I/R injury	Brain	Hu et al. (2005)
Male SD rats	MCAO-induced brain damage	*In vivo and in vitro*	NOX2↓, 8-OHdG↓, 4-HNE↓, 3-NT↓			Sun et al. (2018c)
Primary astrocytes			caspase-3↓, NeuN↓, connexin 43↑			
Wistar rat, Neuronal cells	Neuron damage induced by hydrogen peroxide	*In vitro*	cNOS↓, NO↑	Neuron damage	Brain	Liu et al. (2005)
SD rats	MCAO-induced brain damage	*In vivo*	PKG↑	Cerebral ischemia	Brain	Chen et al. (2021b)
Rat brain microvascular	—	*In vitro*	NO↑, CD63↑, claudin 5↑, occludin↑, ZO1↑, LDH↓, ROS↓	Cerebral ischemia	Brain	Zhong et al. (2019)
Endothelial cells						
Rats	MCAO-induced brain damage	*In vivo*	XOD↓, ALT↓, AST↓, MDA↓	I/R injury	Brain/Liver	Yang et al. (2003)
BV-2 cells, TNC1 astrocytes	MCAO-induced brain damage	*In vitro*	GFAP↑, Notch-1↑, NICD↑, HES-1↑, TNF-α↑, IL-1β↑, iNOS↑	Cerebral ischemia	Brain	Fang et al. (2015)
Male SD rats, BV-2 cells	MCAO-induced brain damage	*In vivo and in vitro*	NF-κB↓, Notch-1↓, NICD↓, RBP-JK↓, Hes-1↓ MCP-1↓	Cerebral ischemia	Brain	Yuan et al. (2015)
Male SD rats	MCAO-induced brain damage	*In vivo and in vitro*	p-p38↓, p-JNK↓, p-ERK1/2↑, iNOS↓, TNF-α↓, IL-1β↓	Cerebral ischemia	Brain	Chen et al. (2020)
BV-2 cells	LPS induced BV-2 cells		p-JNK↓, p-p38 MAPKs↓			
SD rats	MCAO-induced brain damage	*In vivo*	ACE↓, AT1R↓, Ang II↓, TNF-α↓, IL-6↓, IL-1β ↓	Cerebral ischemia	Brain	Wang et al. (2016a)
Male Wistar rats	BCCAO-induced brain damage	*In vivo*	Glu↓, Asp↓, Gly↓, GABA↓, Tau↓, Ca ^2+^-ATPase↑	Cerebral ischemia	Brain	Tang et al. (2014)
			Na ^+^, K ^+^-ATPase↑			
SD rats	—	*In vitro*	p-VASP↑	Hypoxia	Coronary artery	Chen et al. (2015)
SD rats,	Rats with cerebral I/R treatment	*In vivo*	P-VASP↑, PKG↑	Hypoxia	Brai	Du et al. (2015)
HBMECs		*In vitro*				
Male SD rats, BV-2 cell	MCAO-induced brain damage	*In vivo and in vitro*	cyclin B1↑, cyclin B1↑, cyclin D1↑, NT-3↑	Cerebral ischemia	Brain	Fang et al. (2016)
			IGF-1↑, AP-2↑, PSD-95↑			
Male SD rats	—	*In vivo*	eNOS↑, p-Erk5↑, KLF2↑	Subarachnoid hemorrhage	Brain	Li et al. (2016)
Male SD rats	LPS-induced behavioral deficits	*In vivo*	ROS↓NLRP3, caspase-1, IL-1β↓	Depression	Brain	Bian et al. (2020)
Wistar rats	—	*In vivo*	IL-1β↓, IL-6↓, TNF-α↓, SOD↑, MAO↓	Depression	Brain	Guo et al. (2013)
Male C57BL/6 mice	Depression-like behaviors	*In vivo*	TNFα↓, IL-1β↓, IL-6↓, iNOS↓, IL-4↑, BDNF↑	Depression	Brain	Lu et al. (2021)
Primary astrocytes	induced by LPS	*In vitro*				
Male C57BL/6 mice	—	*In vivo*	GABAA Rα1↓, GABAAγ2↓, mEPSC↓	Anxiety disorders	Brain	Guo et al. (2021)
MCF-7 cells	—	*In vitro*	Aggregation of beta-amyloid↓	Alzheimer's disease	Brain	Zhu et al. (2009)
Male APPswe/PS1dE9 mice	—	*In vivo and in vitro*	Aβ aggregation↑, soluble Aβ oligomers↓, Aβ42↓, Aβ40↓	Alzheimer's disease	Brain	Zhang et al. (2020)
C57BL/6 mice						
BV-2 cells	—	*In vitro*	NO↓, TNFα↓, IL-1β↓, ROS↓, iNOS↓, TNFα↓, IL-1β↓	Alzheimer's disease	Brain	Lu et al. (2021)
SH-SY5Y cells			NF-κB↓, JNK↓, p38↓, IFN-γ↓, STAT1α↓			
HT22 cell	—	*In vivo and in vitro*	Lactate dehydrogenase↓, lactate dehydrogenase↓, ROS↓, Aβ1‑42↑	Alzheimer's disease	Brain	Chiba et al. (2012)
Balb/c male mice			p-Tau↓, ROS↓, Bcl-2↑, Bcl-xL↑, Bax↓, cleaved caspase-3↓			
APP/PS1 transgenic mice	—	*In vivo and in vitro*	Aβ plaque↓, TNF-α↓, IL-6↓	Alzheimer's disease	Brain	Zeng et al. (2018)
WT mice, H-SY5Y cell line						
Male SD rats	Permanent bilateral	*In vivo*	Aβ (1-40) ↓, Aβ (1–42) ↓, Iba1↓	Vascular dementia	Brain	Shin et al. (2018)
	Common carotid artery occlusion			Alzheimer's disease		
Male Wistar rats		*In vivo*	nAChR↑, nAChR α4↑, α7↑	Cognitive disorder	Brain	Guo et al. (2011b)
APP/PS1 transgenic mice	—	*In vivo*	SCFAs, IL-1β↓	Alzheimer's disease	Brain	Zhang et al., (2022c)
BALB/cmale mice	—	*In vivo*	MDA↓, SOD↑, IL-1β↓, IL-6↓, HO-1↓, NQO1↑, Nrf2↓	Alcohol induced brain injury	Brain	Zhang et al. (2022d)
BV-2 cells	—	*In vitro*	NF-κB-p65↓, TNF-α↓, IL-1β↓, IL-6↓, NO↓, TNF-α↓, IL-1β↓	neuroinflammation	Brain	You et al. (2018)
			IL-6↓, iNOS↓, IκB↓, IKKβ↓, p38↓, JNK↓, AKT↓			
Male albino Wistar rats	—	*In vivo*	MDA↓, SOD↑, GSH↑, AChE↓, NF-κB↓, TNFα↓, IL-6↓	Cognitive disorder	Brain	Baluchnejadmojarad et al. (2018)
			Nrf2↑, beclin-1↓, LC3 II↓, mTOR↓, P62↓			
Male C57BL/6 mice	—	*In vivo and in vitro*	nestin↑, Tuj-1↑, ERK1/2↑	Cognitive disorder	Brain	Wang et al. (2017)
Neural stem cells						
Male Wistar albino rats	Metanil yellow induced	*In vivo*	GFAP↓, cleaved caspase-3↓, MDA↓, SOD↑, GSH↑	Gliosis	Brain	Tawfeek et al. (2021)
Male SD rats	—	*In vivo*	TLR4↓, NF- κ B p65↓, TNF- α↓, IL-1 β↓, IL-18↓	Hypertension	Brain	Chen et al. (2013)
			Bax↓, cleaved-caspase-3 p17↓, Mcl1↑			
Male SD rats	—	*In vivo*	CTn-T↓, CTn-I↓, AST↓, LDH↓, SOD↑, CAT↑GSH↑	Myocardial infarction	Heart	Huang et al. (2018)
			Caspase3↓, Caspase9↓, cytochrome C↓, NGAL↓, NFκB↓, IL-1β↓			
			P53↓, IL-6↓, Bcl2↑, MDA↓, iNOS↓, Bax↓			
Male Wistar rats	—	*In vivo*	FN1↓, TGFβ1↓, CFs↓, p38-MAPK↓, ERK1/2↓	Cardiac fibrosis	Heart	Pan et al. (2011)
Male SD rats	—	*In vivo*	LVWI↓, RVWI↓, type I and type III collagen↓, MVD↑, CD31↑	Cardiac fibrosis	Heart	Zhou et al. (2014)
			α-sma ↓, Jagged1↑, Notch 1↑, Hes1↑			
C57BL/6 mice	—	*In vitro and in vivo*	CaMKII↓, β-MHC↑, ANP↑	Cardiac hypertrophy	Heart	Pan et al. (2010)
Cardiac myocytes						
SD rats, H9c2 cells	—	*In vitro and in vivo*	NLRP3↓, mTORC1↓, p-Akt↑, Casp-1↓, IL-1β↓	Myocardial I/R injury	Heart	Xu et al. (2020)
Rats, endothelial cell	—	*In vitro and in vivo*	P-JAK↓, P-STAT3↓	Myocardial I/R injury	Heart	Lin et al. (2014)
HCMECs	—	*In vitro*	JAK2↓, p-JAK2↓, STAT3↓, p-STAT3↓	Myocardial ischemia	Heart	Chen et al. (2019)
H9c2 cells	—	*In vitro*	JAK/STAT3↑, Bcl2↑, VEGF↑, MMP2↑, MMP9↑, TNFα↓	Myocardial I/R injury	Heart	Wang et al. (2016b)
			IL-8↓, CK ↓, NO↑, ROS↓, SOD ↑, MDA ↓, STAT3↑, Bcl2↑			
			VEGF↑, MMP2↑, MMP9↑, IL-1β↓, IL-6↓			
HCMECs	—	*In vitro*	EIF6↓, HSPD1↑, CCT6A↑	Anoxia	Heart	Shi et al. (2015)
HCMECs, SD rats	MIR model	*In vitro and in vivo*	PKG-I↑, PKG-Iα↑	Myocardial I/R injury	Heart	Li et al. (2015)
Male SD rats	LPS induced lung injury	*In vivo*	ROS↓, SOD↓, IL-1β↑, IL−18↑, IL−6↑, IL−4↑, IL−10↑, MDA↓	Lung injury	Lung	Fan et al. (2022)
Mice	Injected with a dose of LPS	*In vivo*	TNF-α↓, iNOS↓, c-Fos↓, iNOS↓, NF-kappaB↓, IkBa↓, GSH↑	Lung injury	Lung	Tan et al.(2010)
Male albino rats	Rat Model of Bilateral	*In vivo*	iNOS↑, Bax↑, Bcl2↓, COX2↓	Posterior limb I/R injury	Lung	Ibrahim et al. (2019)
	Hind Limb I/R					
HBE-16 cell		*In vitro*	MUC5AC↓, p-PKC↓, ERK1/2↓	Airway mucus secretion	Lung	Jiang et al. (2011b)
HBE-16 cell, Male SD rats		*In vivo and in vitro*	MUC5AC↓, PKC↓, ERK1/2↓	Airway mucus secretion	Lung	Jiang et al. (2011a)
Male BALB/c mice	BLM-induced	*In vivo and in vitro*	p-p65/p65 ratio↓, IκBα↓NLRP3↓, caspase-1↓, caspase-11↓, IL-1β↓	Pulmonary fibrosis	Lung	Peng et al. (2020)
A549 cell, RLE-6TN cell			IL-18↓, fibronectin↓, vimentin↓, N-cadherin↓, MMP-2↓, MMP-9↓			
HK-2 cells, Wistar rats	—	*In vitro and in vivo*	HO-1↑, SCr↓, BUN↓, KIM-1↓, ROS↓	I/R injury	kidney	Dai et al. (2022)
HK-2 cells	—	*In vitro*	NGAL↓, Kim-1↓, cystatin C↓, IL-18↓, NLRP3↓, CCN1↑	Hyperuricemia	kidney	Li et al. (2020a)
Male C57BL/6 mice		*In vivo*				
Male C57BL/6 mice	—	*In vivo*	BUN↑, CRE↑, TNF-α↓, IL-6↓, Cleaved caspase-3↓	Chemotherapy toxicity	kidney	Sun et al. (2019)
			Cleaved PARP↓, p53↓, Bax/Bcl-2↓, LC3-II/LC3-I↑, Atg7↑			
			p62↓JNK↓, ERK↓, p38↓, stat3↓			
HepG2 cells	Acid-treated HepG2 cells	*In vivo*	XBP1↓, SREBP-1c↓, IRE1α↓	NAFLD	Liver	Zhang et al. (2022e)
C57/BL6 mice	HFD-induced	*In vitro*				
HepG2 cells	—	*In vitro*	CD36↓, Fasn↓, ACC↓, AKT↑, mTOR↓, n-SREBP-1c↓	Hepatocyte lipid metabolism	Liver	Han and Wang (2018)
Male C57BL/6 mice		*In vivo*				
Mice	HFD-induced	*In vivo and in vitro*	SREBP-1c↓, mTOR↓	Hepatocyte lipid metabolism	Liver	Luan et al. (2020)
HepG2 cells	PA-treated HepG2 cells					
Male C57BL/6 mice	HFD induced mice	*In vivo and in vitro*	PPARγ↑, PGC-1α↑, Nrf2↑, HO-1↑, GST↑, NQO1↑	NAFLD	Liver	Zhang et al. (2018)
HepG2 cells	Oleic acid induced cells		NF-κB↓, Keap1↓			
SD rats	HFD-induced	*In vivo*	Nrf2, HO-1, and PI3K, and AKT ↑HO-1, NQO1, and Nrf2↑	NAFLD	Liver	Fan et al. (2017)
	subjected to chronic stress					
BALB/c mice	CCl4-induced liver injury	*In vivo*	AST↓, ALT↓, TBIL↓, IL-1β↓, TNF-α↓, CYP2E1↓, IκBα/NF-κB↓	Hepatotoxicity	Liver	Miao et al. (2021)
S180 tumor-bearing mic	DB-induced liver injury	*In vivo*	MPO↑, IκB↓, NF-κB p65↓, TNF-α↓, IL-6↓, IFN-γ↓, MDA↓, GPx↑	Hepatotoxicity	Liver	Niu et al. (2015)
Male ICR mice	Induced by concanavalin A	*In vivo*	ALT↓, AST↓, TNF-α↓, iNOS↓, c-Fos↓, c-Jun↓, iNOS↓, IkappaB↑	Hepatitis autoimmune	Liver	Tan et al. (2007)
Male Wistar rats	Se-treated rats	*In vivo*	MDA↑, GSH-Px↑, TR↑	Hepatotoxicity	Liver	Eltayeb et al. (2004)
HL-7702	Under hypoxic condition	*In vitro*	ROS↓, MDA↓, SOD↑, bcl-2↑, Keap1↓ Nrf2↑, HO-1↑, NQO1↑, Nrf2↓	I/R injury	Liver	Wu and Jia (2019)
Chondrocytes	—	*In vitro*	MMP1↓, MMP13↓, ADAMTS-5↓, Wnt3a↓, Frizzled7↓	Osteoarthritis	Bones and joints	Liu et al. (2020)
C57BL/6 male mice		*In vivo*	Collagen II↑, Aggrecan↑			
C57BL/6 mice	—	*In vivo and in vitro*	MMP-13↓, ADAMTS-5↓, COX-2↓, iNOS↓, IL-6↓,	Osteoarthritis	Bones and joints	Luo et al. (2020)
Chondrocytes			TNF-α↓, PGE2↓, IL-1β↓, NF-κB↑, Nrf2↑			
BL6/C57 male mice	—	*In vivo and in vitro*	TNF-α↓, IL-1β↓, iNOS↓, MMP13↓, ADAMTS-5↓	Osteoarthritis	Bones and joints	Wang et al. (2019)
Chondrocyte cells			COX-2↓, IL-6↓, NO↓			
SW1353 cells	—	*In vitro*	IL-6↓, AKT↓, mTOR↓, p-mTOR↓, CH25H↓	Osteoarthritis	Bones and joints	Ju et al. (2021)
			CYP7B1↓, ABCA1↑, APOA-1↑			
Human Enucleus	—	*In vivo and in vitro*	ROS↓, NF-κB↓, MAPK↓, TNF-α↓, NLRP3↓	Intervertebral	Bones and joints	Wang et al. (2022b)
Pulposus Cells				disc degeneration		
Male SD rats						
SD rats	—	*In vivo and in vitro*	ATG5↑, Rab8a↑, PI3K↑, PTEN↑, Akt↑	Intervertebral	Bones and joints	Hu et al. (2022)
Nucleus pulposus cells				disc degeneration		
Male DBA/1J mice	collagen‑induced arthritis	*In vivo*	IL‑1β↓, IL‑6↓, TNF‑α↓, Caspase‑3/-9↓Bax/Bcl‑2↓TLR4↓NF‑κB↓	Arthritis	Bones and joints	Zhang et al., (2017)
Raw264.7 cell line	—	*In vivo and in vitro*	RANKL↓, MAPK↓, NF-κB↓, JNK1/2↓, p38↓, ERK1/2↓, IκBα↓	Arthritis	Bones and joints	Zhao et al. (2016)
C57BL/6 male mice						
Osteoblasts female SD rats	—	*In vivo and in vitro*	ALP secretion↑, intracellular calcium ion influx↑	Osteoporosis	Bones and joints	Wang et al. (2018)
			calcium deposition↑, CXCR4↑, p65↑			
3T3-L1 cells		*In vitro*	PPARγ↓ C/EBPα↓	Obesity	Metabolic disease	Lu et al. (2013)
db/db mice db/m^+^ mice	—	*In vivo*	Nrf2↑, HO-1↑, IL-1β↓, IL-2↓, IL- 4↑	Diabetic Nephropathy	Diabetic complication	Liu et al. (2019b)
C57BL/6 male mice	—	*In vitro and in vivo*	laudin-1↑, claudin-19↑, NFκB↓, TNF-α↓, p-ERK 1/2↓, Nrf2↑	Diabetic retinopathy	Diabetic complication	Mei et al. (2019)
WT mice and HRECs						
Human retinal endothelial cells	—	*In vitro*	VEGF↓, p-ERK↓, p-FAK↓, p-Src↓	Diabetic retinopathy	Diabetic	Long et al. (2019)
Rats		*In vivo*			complication	
Male Wistar rats	—	*In vivo*	MDA↓, ROS↓, Bcl-2↑, BAX↓, VEGF↑	Testicular Damages	Diabetic	Long et al. (2015)
					complication	
Male SD rats	—	*In vivo*	blood glucose↓, TC↓, TG↓ LDL↓, HDL↑, LDH1↓, CK↓,	Diabetic cardiomyopathy	Diabetic	Su et al. (2022)
			Beclin-1↑, LC3-II↑		complication	
Male Swiss mice	HFD-induced	*In vivo*	CK-MB↓, Troponin↓, BNP↓, SOD↑, CAT↑, GPx↑, GST↑, Nrf2↓	Diabetic cardiomyopathy	Diabetic	Huo et al. (2021)
			Nqo-1↓, Ho-1↓, Tlr4↓, Myd88↓, Nf-κb↓, IL-6↓, TNf-α↓, IKKβ↑		complication	
			Cyt-c↓, Parp 1↓, bcl-2↑, caspase-3↓, caspase-9↓, Bax↓			
HUVECs	—	*In vitro*	Bcl-2↓, Bax↓ Cyt c↓, ROS↑, SOD↑, SOD2↑, LC3 II↑, Beclin 1↑	Diabetic	Diabetic	Xi et al. (2021)
			Atg 5↑, PINK1↑, Parkin↑, Mitofusin2↑	cardiomyopathy	complicatio	
Male C57/B6 mice	—	*In vivo*	NLRP3↓, NF-κB↓, p-AKT↓, Nrf2↓, HO-1↓, EF↓, LVVd↓	Diabetic	Diabetic	Xu et al. (2021)
			CK↓, Col I↓, TGF-β1↓, SOD↑, CAT↑, GSH-Px↑, MDA↓	cardiomyopathy	complication	
			ROS↓, IL-1β↓, IFN-γ↓, IL-6↓, MCP-1↓, TNF-α↓, IL-18↓			
			LDH↓, cTnI↓			
LO2	Induced by Hcy	*In vivo and in vitro*	Hcy↓, TG↓, CHO↓, LDL↓, ALT↓, AST↓, insulin↓, CBS↓, CSE↓	Diabetic	Diabetic	Wang et al. (2020)
Male SD rats	High-fat induced		MTHFR↓, folic acid↑, VitB6↑, VitB12↑	Liver Injury	complication	
PC-9, H1975, Hela cells	—	*In vitro*	p-ERK1/2↑ ERK1/2↓, p-AKT↓, AKT↓, LC3-II↓	Lung cancer	Cancer	Sun et al. (2018a)
HepG2, Beas-2B cells			p-ERK1/2↑, p-AKT↓			
A549 cells	—	*In vitro*	G0/G1↓, AKT↓, mTOR↓, BCL-XL↓, STAT3↓, p-STAT3↓, 4EBP1↑	Lung cancer	Cancer	Cao et al. (2019)
A549 cells	—	*In vitro*	ROS↑, caspase-3↑, TGF-β1↓	Lung cancer	Cancer	Zhang et al. (2021)
HepG2 and MHCC97-H cells	—	*In vitro*	EMT↓, p-JAK2↓, p-STAT3↓ E‐cadherin↑, snail↓, vimentin↓	Liver cancer	Cancer	Liu et al. (2019a)
HepG2 and SK-Hep1 cells	—	*In vitro*	STAT3↓, Girdin↓, AKT↓	Liver cancer	Cancer	Ke et al. (2017)
Male BALB/c nude mice		*In vivo*				
C57BL/6 male mice	CAC caused by AOM/DSS	*In vivo and in vitro*	NF-κB↓, SHH↓, Ptch1↓, Smo↓, Gli1↓	Colorectal cancer	Cancer	Zeng et al. (2022)
SW480 cells						
SW620, HCT116, LOVO	—	*In vitro and in vivo*	ephrinb2↓, ephB6↓, ephA1↓	Colorectal cancer	Cancer	Zhu et al. (2017)
HT29 cells and Balb/c nude mice						
HT-29 CSC cells	—	*In vitro and in vivo*	Lgr5↓, c-Myc↓, CK20↓, Nanog↓, Gli1↓, CD133↓, Lgr5↓	Colorectal cancer	Cancer	Lei et al. (2020)
Nude mice			Gli1↓, Ptch1↓, c-Myc↓, Ki-67↓, CK20↓			
HCT116 p53^+/+^ (wild-type)	—	*In vitro*	caspase-6↑	Colorectal cancer	Cancer	Chan et al. (2009)
p53^-/-^ (knockout) cells						
Male C57BL/6 mice	Induced by azoxymethane and	*In vivo*	Wnt/β-catenin↓, TNF-α↓, IL-6↓, Bax↑	Colorectal cancer	Cancer	Zeng et al. (2021)
HT-29 cells	dextran sulfate sodium	*In vitro*	Bcl-2↓, GSK-3β↓, cyclin D1↓			
Mice	—	*In vivo*	p38 MAPK↓, TNFR2+Tregs↓, CD8+T↑	Colorectal cancer	Cancer	Chen et al. (2022)
WEHI-13VAR and CT26 cell		*In vitro*				
HCT‑116 cells	—	*In vitro*	Bcl‑2↓, Bax↑, caspase‑3↑, p-p53↑	Colorectal cancer	Cancer	Yang et al. (2017)
AGS	—	*In vitro and in vivo*	LDH↓, G0-G1↑S↓, G2-M↓, SOD↑, GSH↑, CAT↑, MDA↓, 8-OHdG↓	Gastric cancer	Cancer	Sun and Meng (2022)
MGC-803 and AGS	—	*In vitro*	PI3K↓, PTEN↑	Gastric cancer	Cancer	Li et al. (2021)
SAS cells	—	*In vitro and in vivo*	MMP-2 and -9↓, integrin αvβ6↓, c-JUN↓	Oral squamous cell carcinoma	Cancer	Li et al. (2013)
Athymic Balb/ca nude mice						
SAS and HSC-4 cells	—	*In vitro*	E-cadherin↑, αvβ6 integrin↓	Oral squamous cell carcinoma	Cancer	Li et al. (2010)
C57BL/6J mice	—	*In vivo*	retinal thinning and reduced visual behavioral deficits	Glaucoma	Eye	Pang and Clark (2020)
Female C57BL/6 mice	LPS-primed macrophages	*In vivo and in vitro*	NLRP3↓, IL-1β↓, caspase-1 ↓	Sepsis	Multiple Organ	Liu et al. (2017)

### 2.1 Cerebrovascular diseases

#### 2.1.1 Cerebral ischemia/reperfusion injury

**FIGURE 3:**
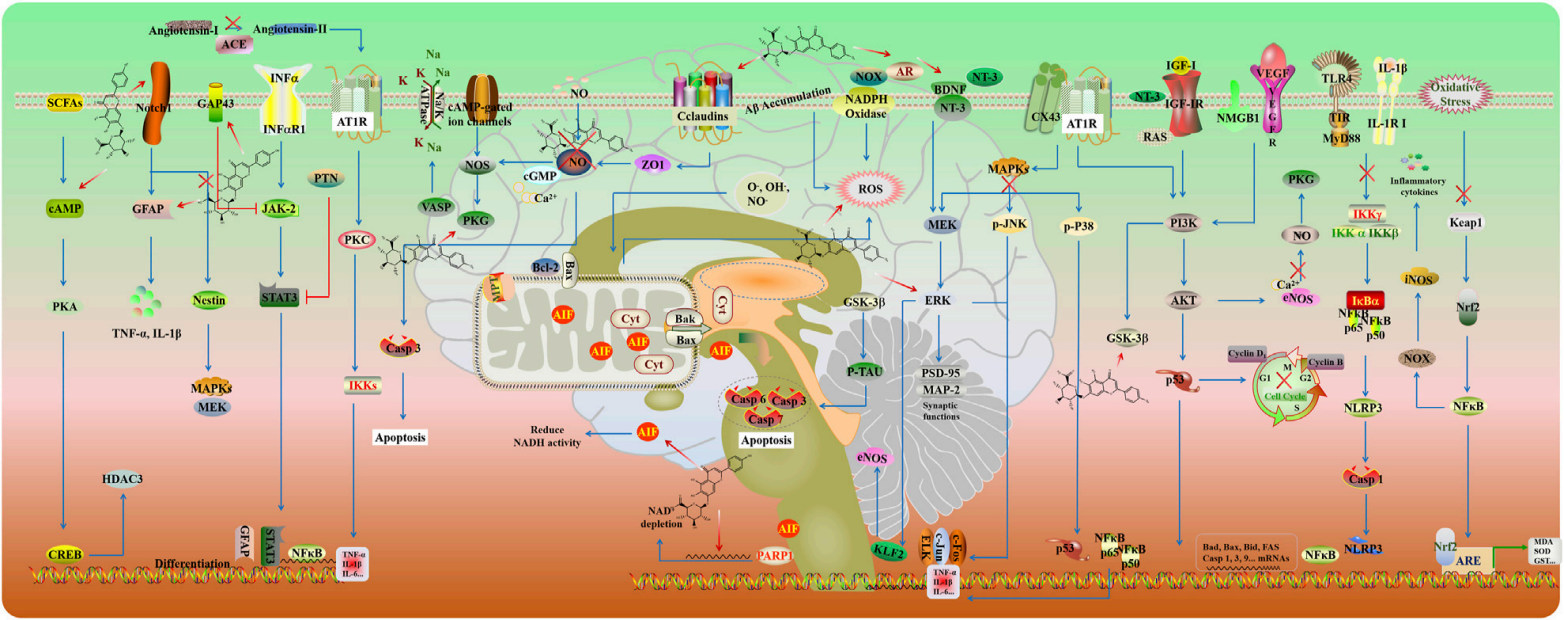
Potential mechanisms and targets of cerebral protection effect.

Cerebral ischemia/reperfusion injury (CIRI) refers to brain damage that occurs when blood supply is restored to the brain following a period of ischemia. The primary treatment involves timely thrombolytic therapy or surgical intervention (Zhang et al., 2024). However, reperfusion can potentially promote secondary cell death and exacerbate brain injury, leading to cerebral ischemia/reperfusion injury (Zheng et al., 2023). In the initial stage of ischemia, insufficient blood flow during cerebral ischemia results in an inadequate supply of glucose, and oxygen, low ATP, and excessive glutamate excitatory toxicity (Huang et al., 2023). Consequently, excessive release of glutamate leads to calcium (Ca^2+^) overload and further triggers the generation of free radicals and nitric oxide (NO), initiating a cascade of detrimental processes. These include mitochondrial dysfunction and DNA damage, which collectively promote oxidative stress and neurotoxicity. Upon reperfusion, the accumulation of reactive oxygen species (ROS) and inflammatory cells, such as neutrophils, exacerbates the ischemic damage. These pathological processes are associated with oxidative stress, destruction of the blood-brain barrier, inflammation, apoptosis, and ionic imbalance (Candelario-Jalil et al., 2022; Oyefeso et al., 2021). The signal transduction pathways involved in CIRI are summarized in Figure 3. Therefore, it is imperative to explore novel drugs that target the underlying pathological progression of CIRI, to enhance neurological recovery and prognosis in patients. The action mechanism of SCU protected against CIRI mainly includes anti-apoptosis, anti-oxidative stress, anti-inflammatory, and regulation of the ion channel.

Increasing evidence suggests that cerebrovascular disease is closely linked to multiple forms of programmed cell death (PCD), such as apoptosis, autophagy, pyroptosis, and ferroptosis. Consequently, the targeted inhibition of these PCD pathways plays a critical role in mitigating the severity of cerebrovascular diseases and improving neurological outcomes (Zhang et al., 2022a). NO and tumor necrosis factor (TNF-α) activate intrinsic and extrinsic pathways of apoptosis in CIRI. Emerging evidence indicates that apoptosis involves the synthesis of new proteins (Gong et al., 2017). Poly (ADP-ribose) polymerase (PARP) is a DNA-binding protein that utilizes nicotinamide adenine dinucleotide (NAD) as a substrate and is activated by extensive DNA damage (Chatterjee et al., 2022). PARP-1 produces long and branched poly-ADP ribose (PAR) polymers. PAR production and translocation to the cytosol induces a cascade of events, including PAR binding to mitochondrial apoptosis-inducing factor (AIF), AIF translocation to the cytosol, AIF binding to macrophage migration inhibitory factor (MIF), co-translocation of AIF-MIF complex to the nucleus, and large-scale DNA fragmentation by MIF nuclease activity. These steps result in subsequent cell death (Yang et al., 2024a).

In a rat model of middle cerebral artery occlusion (MCAO), SCU reduced the infarct volume and ameliorated the neurological deficit by inhibiting PARP overactivation and AIF translocation from the mitochondria to the nucleus following CIRI (Zhu et al., 2009). Moreover, SCU plays a neuroprotective role by reducing microglial neuroinflammation and apoptosis mediated by the activated PI3K/AKT/GSK3β/NF-κB signaling pathway in LPS-induced BV2 cell (Duan et al., 2024). In oxygen-glucose deprivation and reperfusion-induced HT22 cell injury, SCU pretreatment could improve mitochondrial dysfunction and inhibit apoptosis by stimulating mitophagy (Yang et al., 2024c).

Neonatal hypoxic-ischaemic encephalopathy (HIE) is a major cause of neonatal mortality due to its devastating impact on neonatal brain development (Greco et al., 2020). Increasing evidence indicates that HIE can lead to acute cerebral reperfusion injury, edema, increased intracranial pressure, impaired autoregulation, and hemorrhage, which are known as important pathologies of later neurodevelopmental impairments (Cao et al., 2020). Growth-associated protein 43 (GAP43), a nervous tissue-specific cytoplasmic protein, plays a crucial role in neurite outgrowth during axon development and regeneration. Inhibiting GAP43 expression exerts adverse effects on axon outgrowth (Dan et al., 2022). SCU treatment could improve cell viability, and ameliorate cell apoptosis and long-term neurological deficits after HI injury via upregulating GAP43 expression and inhibiting JAK/STAT3 signaling in oxygen-glucose deprivation-induced primary cortical neurons (Niu et al., 2021).

Reactive oxygen and nitrogen species (ROS/RNS) are continuously produced from internal metabolism and external exposures in mammalian systems. ROS/RNS in physiological amounts serve as mediators and regulators, ensuring proper cellular functions: growth, proliferation, differentiation, and apoptosis. However, the imbalance between the continuous production of reactive oxygen species and their elimination as a result of enzymatic and non-enzymatic neutralization reactions and the action of exogenous antioxidants causes oxidative stress, which leads to brain damage after a stroke and permanent or reversible neurological deficits (Pawluk et al., 2024). Due to hypoxia, there is a deficit of ATP, a decrease in energy, an influx of calcium, and, as a result, mitochondrial failure. Excitotoxicity and ROS/RNS activity stimulate nerve cells, mainly microglia and astrocytes, to secrete inflammatory markers. Increased activity of pro-inflammatory cytokines generates ROS, which are responsible for protein oxidation, peroxidation of polyunsaturated fatty acids, and disruption of redox homeostasis, ultimately leading to cell death (Wierońska et al., 2021). In the CIRI rat model, SCU is an efficient radical scavenger against ROS and RNS, including hydroxyl radical, superoxide anion radical, and hydrogen peroxide (Zhang Y. et al., 2022).

Nuclear factor erythroid 2-related factor 2 (Nrf2) is a vital transcription factor that regulates antioxidant defense and detoxification enzymes, including NAD(P)H quinone oxidoreductase, heme oxygenase-1 (HO-1), and glutathione S-transferases (GSTs) (He et al., 2020). SCU showed antioxidant activity by promoting Nrf2 nuclear translocation, upregulating HO-1 expression, increasing superoxide dismutase (SOD) activity, and inhibiting ROS generation in OGD/R-induced HT22 cells. Furthermore, SCU reduced infarct volume and blood-brain barrier (BBB) permeability, improved sensorimotor functions and depressive behaviors, and alleviated oxidative stress and neuroinflammation by activating PI3K/Akt/Nrf2 signaling in CIRI rats (Zhang Y. et al., 2022).

Aldose reductase (AR) is a key protein in the polysaccharide pathway of sugar metabolism that regulates the intracellular redox balance and maintains cellular osmotic pressure and oxidative stress (Sardelli et al., 2023). In the CIRI rat model, SCU remediated oxidative stress injury by activating AR- NADPH oxidase (NOX) (Deng et al., 2022).

NO is an important signaling molecule that plays a key role in the central nervous system (CNS) (Iova et al., 2023). During ischemia, NO reacts with ROS, resulting in the formation of reactive radicals. Chen Y. J. et al. (2021) demonstrated that SCU has neuroprotective properties by activating NO synthase (NOS) and protein kinase G (PKG). Moreover, SCU pretreatment could ameliorate the neurological deficit and reduce the permeability of the BBB by upregulation of eNOS expression and downregulation of VEGF, bFGF, and iNOS expression after CIRI (Wang et al., 2021). The loss of connexin 43 (CX43), a gap junction protein in astrocytes, can exacerbate neuronal injury in cerebral ischemia (Yin et al., 2018). Sun J. B. et al. (2018) suggested SCU alleviates brain ischemic injury by regulating the expression of NOX2 and CX43.

Homocysteine (Hcy) is an important risk factor for stroke, whose overexpression reduces the ability of tight junction (TJ) proteins and alters the basement membrane, destroying the BBB. Exosomes have been shown to accelerate functional recovery and neurovascular plasticity following ischemia by modulating TJ proteins (Huang et al., 2022). SCU-treated exosomes could enhance cell viability of homocysteine-induced rat brain microvascular endothelial cells by increasing the expression of NO, claudin 5, occludin, and zonula occludens-1 (ZO-1) and decreasing the expression of LDH and ROS (Zhong et al., 2019).

Inflammation is a key factor in the pathogenesis of ischemic stroke (Chen J. et al., 2021). Anti-inflammatory therapy is a potential therapeutic strategy for post-CIRI. Accumulating evidence has shown that SCU exerts neuroprotective effects by modulating multiple inflammatory signaling. Neuroinflammation contributes to the progression of cerebral ischemia/reperfusion (I/R) damage. The Notch pathway plays a vital role in activated microglia in response to hypoxic brain injury through its transactivation of NF-κB and subsequent cytokine release. In the CIRI rat model and LPS-induced BV-2 cells, SCU attenuated microglia-mediated neuroinflammation by inhibiting the Notch/NF-κB pathway (Xie et al., 2019; Yuan et al., 2015). Moreover, the NF-κB signaling pathway is positively controlled by MAPK which is another regulator that controls the production and release of pro-inflammatory factors in response to cerebral ischemic injury (Xie et al., 2019). SCU could protect the brain against neuroinflammatory injuries by inhibiting the MAPK/NF-κB signaling in CIRI rats (Zhang Y. et al., 2022).

Angiotensin-converting enzyme (ACE) of the renin-angiotensin system plays an important role in stroke (Abdel-Fattah et al., 2018). ACE, which converts angiotensin I (Ang I) into angiotensin II (Ang II), is closely linked to brain edema, inflammatory response, and neuronal apoptosis following ischemic stroke. Ang II, after binding to the Ang II type 1 receptor (AT1R), can induce ischemic injury by causing local cerebrovascular vasoconstriction and dysfunction. SCU decreased neurological deficit score, infarct area, and cell apoptosis in CIRI rats by inhibiting the Ang II/AT1R pathway in a dose-dependent manner (Wang W. et al., 2016).

During the CIRI, the disruption of blood deprives cells of energy and disturbs the ionic homeostasis of the cells. Brain edema is a typical syndrome in ischemic cerebrovascular disease, partly resulting from the dysfunction of Na^+^ and K^+^-ATPase in the cell membrane. Glutamate receptor-mediated ionic imbalance and neurotoxicity have been well-established in cerebral ischemia (Olivares-Bañuelos et al., 2019). SCU could attenuate neuronal cell damage and reduce brain edema by regulating the levels of glutamic acid, aspartic acid, and gamma-aminobutyric acid (GABA). Additionally, SCU increased the activities of Ca^2+^-ATPase and Na^+^, K^+^-ATPase (Tang et al., 2014).

Accumulating evidence indicates that PKG dysfunction is related to CIRI. Vasodilator-stimulated phosphoprotein (VASP), an important PKG-I substrate and actin regulatory protein, serves as a critical indicator of PKG-I activity and downstream ion channels in intact cells (Xu et al., 2023). SCU enhanced endothelium-dependent relaxation in isolated basilar arteries and counteracted vascular endothelium dysfunction in hypoxia-reoxygenation-induced human brain microvascular endothelial cells (HBMECs) by increasing the expression of VASP (Du et al., 2015). Moreover, SCU promotes the production of neurotrophic factors and accelerates neuronal integrity and synaptic plasticity of microglia by upregulation of the expression of brain-derived neurotrophic factor (BDNF), such as neurotrophin 3 (NT-3), insulin-like growth factor-I (IGF-I), microtubule-associated protein-2 (MAP-2) and postsynaptic density protein-95 (PSD-95) (Fang et al., 2016).

#### 2.1.2 Subarachnoid hemorrhage and behavioral deficits

Subarachnoid hemorrhage (SAH) is a neurological disease with high morbidity and mortality. Dysfunction of eNOS plays an indispensable role in vasospasm post-SAH (Gao et al., 2022). Kruppel-like factor 2 (KLF2) is a key regulator of eNOS, affecting vascular tone, inflammation, and cell migration. Extracellular-regulated kinase 5 (Erk5) modulates KLF2 and eNOS (Angolano et al., 2021). SCU improved SAH by increasing the expression of eNOS in the intima of the cerebral arteries and enhanced the levels of p-Erk5 and KLF2 (Li et al., 2016).

Depression is a complex mental disorder linked to inflammatory reactions and microglial activation and affects approximately 350 million people worldwide (Wang H. et al., 2022; Xia et al., 2023). Activation of the Nod-like receptor pyrin-containing pyrin domain 3 (NLRP3) inflammasome in microglia leads to caspase-1 activation and subsequent production of bioactive IL-1β from pro-IL-1β. ROS promotes tissue inflammation and immune response via the NLRP3 pathway. SCU ameliorated LPS-induced depressive-like behaviors by inhibiting ROS generation and decreasing the expression of NLRP3, caspase-1, and IL-1β (Bian et al., 2020). Another prevalent psychiatric symptom is anxiety, which significantly impacts daily life and is related to glutamate neurotransmission. GABA, an inhibitory neurotransmitter, counteracts glutamate’s excitatory effects. SCU exhibited protective effects against anxiety-like behavior by downregulating glutamatergic receptors and abrogating GABA_A_ Rα1 and GABA_A_ γ2 in the prefrontal cortex (Guo et al., 2021).

#### 2.1.3 Neurodegenerative disease

Alzheimer’s disease (AD) is a prevalent neurodegenerative condition characterized by amyloid formation, neurofibrillary degeneration, and synaptic loss. β-amyloid peptide (Aβ), derived from amyloid precursor protein (APP) cleavage by β- and γ-secretases, is central to cognitive dysfunction in AD (Jucker and Walker, 2023). SCU has demonstrated inhibitory effects on the aggregation of Aβ, reducing high toxic soluble Aβ 42 and Aβ 40 levels while elevating less toxic amyloid plaques in the cortex (Hu et al., 2018; Shin et al., 2018; Zeng et al., 2018; Zhang et al., 2020). Oxidative stress and inflammation contribute to AD progression by increasing the aggregation of Aβ (Zeng et al., 2018; Zhang S. et al., 2022; Zhang T. et al., 2022). SCU improved cognitive impairments in AD mice by upregulating the expression of Aβ-42 deposition and phosphorylated-Tau in the hippocampus of AD mice. Additionally, SCU enhanced SOD and GSH levels while reducing the levels of inflammatory factors such as iNOS, TNF-α, IL-1β, and IL-6 by inhibiting NF-κB signaling (Baluchnejadmojarad et al., 2018; Guo L. L. et al., 2011; Hu et al., 2018; Wang et al., 2017; You et al., 2018).

Neuronal nicotinic acetylcholine receptors (nAChRs) are recognized as therapeutic targets for improving cognitive function and retarding neurodegeneration in AD (Hoskin et al., 2019). SCU alleviated behavioral deficits by increasing the expression of α4 and α7 nAChR subunit and restoring the activities of acetylcholinesterase (AChE) and butyrylcholinesterase (BuChE) in AD mice (Guo L. L. et al., 2011). Additionally, the potential neuroprotection mechanism of SCU is partly attributed to the inhibition of p38 MAPK signaling (Wang et al., 2017; You et al., 2018).

### 2.2 Cardioprotective effects

**FIGURE 4:**
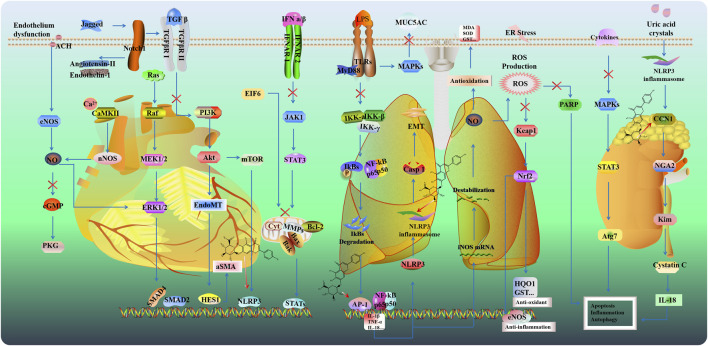
Potential mechanisms and targets of cardioprotection, lung and kidney protection.

Hypertension is a major risk factor for cardiovascular events, such as ischemic stroke and cerebral hemorrhage, primarily due to its role in inflammation-mediated target organ damage. SCU reduced inflammatory responses in renal artery constriction-induced hypertension rat model by suppressing TLR4/NF-κB signaling and apoptotic markers like Bax and cleaved-caspase-3 (Mehta et al., 2014). Additionally, SCU demonstrated vasodilatory effects in isolated blood vessels by relaxing the thoracic and abdominal aortas in an endothelium-dependent manner (Chen Y. J. et al., 2021). Cardiovascular disease (CVD) poses a serious threat to patients’ physical and mental health, as well as their quality of life, due to its high morbidity and mortality rates. SCU offers multiple cardiovascular benefits, including anti-myocardial fibrosis, protection of vascular endothelial function, reduction of myocardial injury, and cardiac function. In cardiovascular diseases, SCU exhibits cardioprotective effects attributed to its actions against oxidative stress, inflammation, apoptosis, and fibrosis. These mechanisms contribute to its therapeutic benefits in mitigating cardiovascular morbidity and mortality (Figure 4).

#### 2.2.1 Myocardial infarction

Myocardial infarction (MI) is related to the imbalance between coronary blood supply and myocardial demand, leading to cardiac remodeling and chronic heart failure. Loss of cardiomyocytes during either the acute or chronic stage of MI directly contributes to contractile dysfunction. Circulating apoptotic markers soluble TNF receptor 1 (sTNFR1) and sTNFR2 were found to be associated with myocardial infarct size and left ventricular insufficiency in patients with ST-segment elevation myocardial infarction (STEMI), suggesting that apoptosis may be a key determinant of the extent of I/R injury (Zhang et al., 2022b). SCU improved the impaired cardiac function of infarct rats and decreased interstitial fibrosis by downregulating pro-apoptotic markers such as Bax, caspase-3, caspase-9, and p53, while upregulating the anti-apoptotic protein Bcl-2 in an isoprenaline-induced rat model of MI (Huang et al., 2018; Rodríguez et al., 2002).

Fibrosis, another hallmark of post-MI remodeling, contributes significantly to the progression of ventricular function. Transforming growth factor-β1 (TGFβ1) is an indispensable molecule in cardiac fibrosis (Li et al., 2018). Activation of Notch signaling could restrain TGF-β induced EndoMT and myocardial fibrosis (Dong et al., 2023). SCU significantly improved cardiac function by inhibiting interstitial fibrosis, and the mechanisms may involve the suppression of pro-fibrotic cytokine TGFβ1 expression, inhibition of p38 MAPK and ERK1/2 phosphorylation, and activation of Notch signaling (Pan et al., 2011; Zhou et al., 2014). Intracellular calcium overload is involved in the pathogenesis of cardiac hypertrophy following MI. SCU exerts anti-hypertrophic effects by inhibiting the calcineurin-NFAT and CaMKII pathways (Rostas and Skelding, 2023).

#### 2.2.2 Myocardial ischemia-reperfusion injury

Myocardial ischemia-reperfusion injury (MIRI), occurs during the restoration of blood flow to the ischemic myocardium and exacerbates cardiac dysfunction. SCU exerted a role in inhibiting NLRP3 activation and thus attenuating the inflammatory response by increasing AKT phosphorylation, and inhibiting mTORC1 activity in experiments in which acute myocardial I/R injury induced H9c2 damage. Moreover, SCU exerted cardioprotective effects in the experiments on I/R-injured H9c2 through the JAK/STAT3 signal pathway (Chen et al., 2019; Shen et al., 2021; Xu et al., 2020). The eNOS-cGMP-PKG pathway is considered a target for attenuating IR injury (James et al., 2023). In an experimental model of MIRI in rats, SCU restored endothelium-dependent vasodilation by increasing PKG-Iα levels, and pVASP protein and further improving the responsiveness of coronary artery rings to acetylcholine (Li et al., 2015).

### 2.3 Pulmonary protective effects

Acute lung injury (ALI) is a severe pulmonary disease characterized by pulmonary edema and increased alveolar permeability. Excessive lung inflammation heightened neutrophil infiltration, increased microvascular permeability, interstitial edema, thickened alveolar walls, and impaired gas exchange, all of which contribute to significant respiratory dysfunction (Sharawi et al., 2024). Mitochondrial dysfunction aggravates the deterioration of lung function by promoting excess ROS (Figure 4). In the LPS-induced ALI model of rats, SCU pretreatment reversed the high levels of ROS and MDA while increasing the levels of SOD and GSH via the inhibition of the JNK/c-jun/Phospho-c-jun/cleaved caspase three signaling pathway (Fan et al., 2022). Additionally, SCU decreased the expression of inflammatory cytokines such as IL-1β, IL-18, IL-6, and TNF-α in bronchoalveolar lavage fluid via inhibiting activator protein 1 (AP-1) and NF-κB signaling (Ibrahim et al., 2019).

Chronic airway diseases are characterized by persistent mucus hypersecretion and inflammation, leading to respiratory dysfunction. In human neutrophil elastase-induced rats and cell models, SCU treatment inhibited mucus hypersecretion in a concentration-dependent manner via inhibition of the expression of mucin 5AC and the phosphorylation of PKC and ERK1/2 (Jiang et al., 2011a). In another study, SCU suppressed inflammation and inflammatory cell infiltration into the lungs and attenuated airway hyperresponsiveness and airway remodeling in ovalbumin-challenged asthmatic mice. Moreover, SCU prevented the TGF-β-induced migration and EMT in 16HBE cells. The potential mechanism is related to the inactivation of the Smad/MAPK and NF-κB/NLRP3 pathways (Li et al., 2024; Peng et al., 2020).

### 2.4 Kidney and liver protective effects

Kidney injury (AKI) results in high morbidity and mortality among inpatients, while effective treatment and intervention are still absent. Inflammatory response, apoptosis, and oxidative stress play key roles in the pathogenesis of kidney injury (Li et al., 2024) (Figure 4). SCU protected renal tubular function against renal ischemia-reperfusion injury and increased the expression of antioxidant enzymes (SOD, CAT, HO-by activating the Nrf2/ARE signaling pathway (Dai et al., 2022). NLRP3 could be activated by uric acid crystals and increased IL-1β. SCU dose-dependently alleviated the renal injury, and apoptosis by downregulating the expression of NLRP3, IL-1β, NGAL, Kim-1, cystatin C, and IL-18 and increasing anti-apoptosis CCN1 in hyperuricemic nephropathy mice (Ding et al., 2022; Li G. et al., 2020). Additionally, SCU protected against cisplatin-induced renal injury by inhibiting MAPK pathways, reducing the Bax/Bcl-2 ratio, and suppressing cleaved caspase-3 and PARP cleavage and Atg7-dependent autophagy (Zhang X. et al., 2022).

**FIGURE 5:**
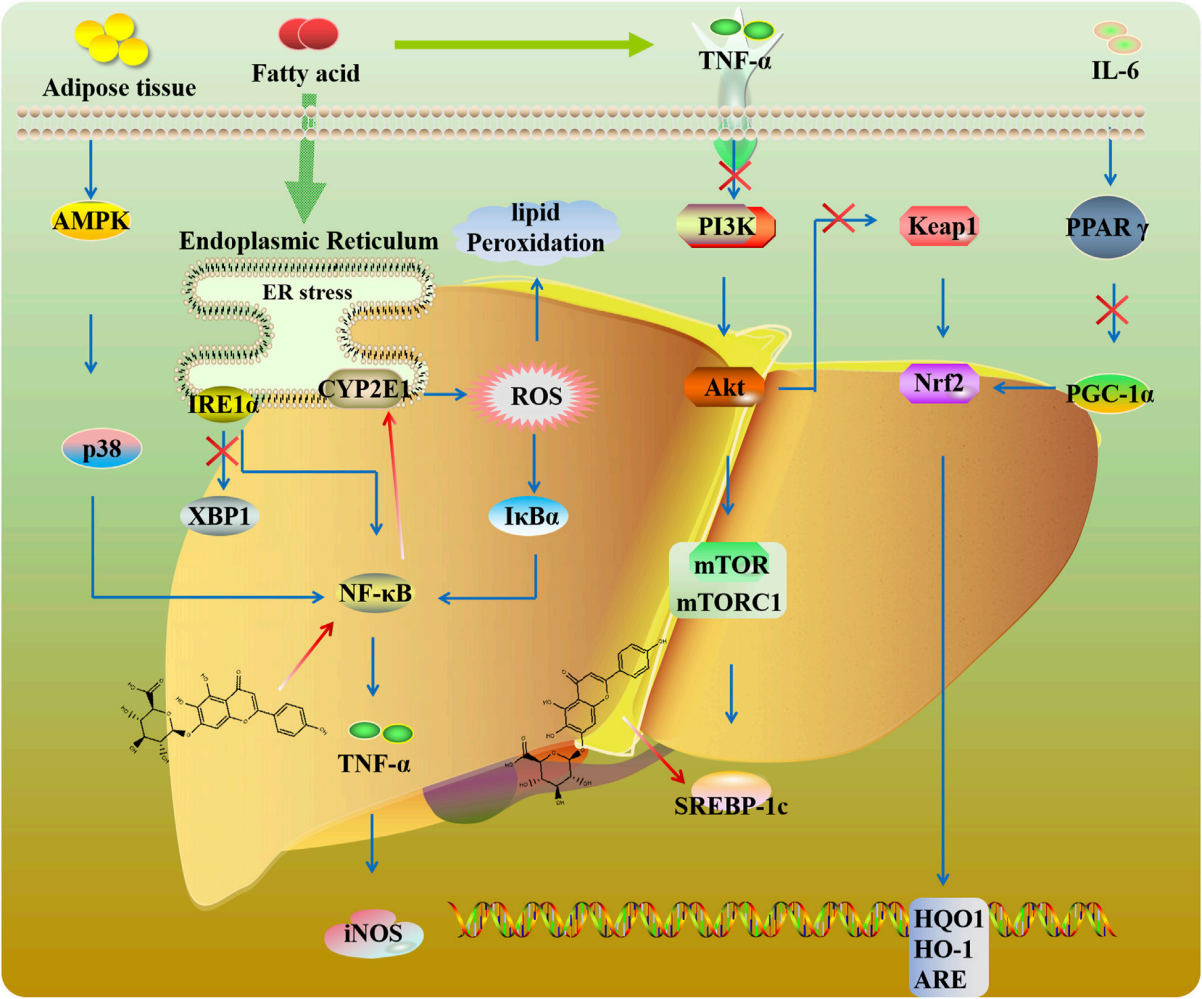
Potential mechanisms and targets of liver protection.

Nonalcoholic fatty liver disease (NAFLD) is the most prevalent liver disease, characterized by the presence of steatosis in more than 5% of hepatocytes with little or no alcohol intake. Endoplasmic reticulum (ER) stress is related to the progression of NAFLD. Sun et al. (2019) found that SCU mitigated hepatic lipid accumulation by inhibiting inositol-requiring enzyme 1α (IRE1α)/X-box-binding protein 1 (XBP1) signaling and further suppressing ER stress (Figure 5). Hepatic lipid accumulation activates PI3K/AKT/mTOR signaling (Han and Wang, 2018). mTORC1 could promote sterol-regulatory element binding protein (SREBP)-dependent lipogenesis. In high-fat diet (HFD) mice, SCU could ameliorate insulin resistance via mTOR/SREBP-dependent pathway (Han and Wang, 2018; Luan et al., 2020). Peroxisome proliferator-activated receptor gamma (PPARγ) plays a meaningful role in adipocyte differentiation and inflammation. As a transcriptional coactivator of PPARγ, peroxisome proliferator-activated receptor gamma coactivator-1 alpha (PGC-1α) is involved in mitochondria generation. It has been reported that PPARγ binds to the Nrf2 promoter and regulates the expression of antioxidant genes (Li L. et al., 2020). SCU exerts hypolipidemic, antioxidative, and liver protective by regulating the PPARγ/PGC-1α-Nrf2 signaling pathway (Zhang et al., 2018).

Toxicity to hepatocytes caused by various insults including drugs is a common cause of chronic liver failure. CYP2E1 biotransforms toxins like carbon tetrachloride (CCl_4_) into hepatotoxins, exacerbating liver injury. SCU improved lipid metabolism and bile acid homeostasis by regulating CYP2E1 and NF-κB signaling in mice exposed to CCl4 (Miao et al., 2021). Moreover, SCU could alleviate poisoning-induced liver injury like selenium, concanavalin A, and diosbulbin B by regulating the NF-κB-TNF-α-iNOS pathway (Eltayeb et al., 2004; Niu et al., 2015; Tan et al., 2007). Liver I/R injury is a common complication after liver transplantation, stroke, and trauma (Liang et al., 2022). SCU protects the liver against oxidative stress by mediating Keap1/Nrf2/ARE signaling in I/R-induced hepatocytes (Wu and Jia, 2019).

### 2.5 Orthopedic diseases

**FIGURE 6:**
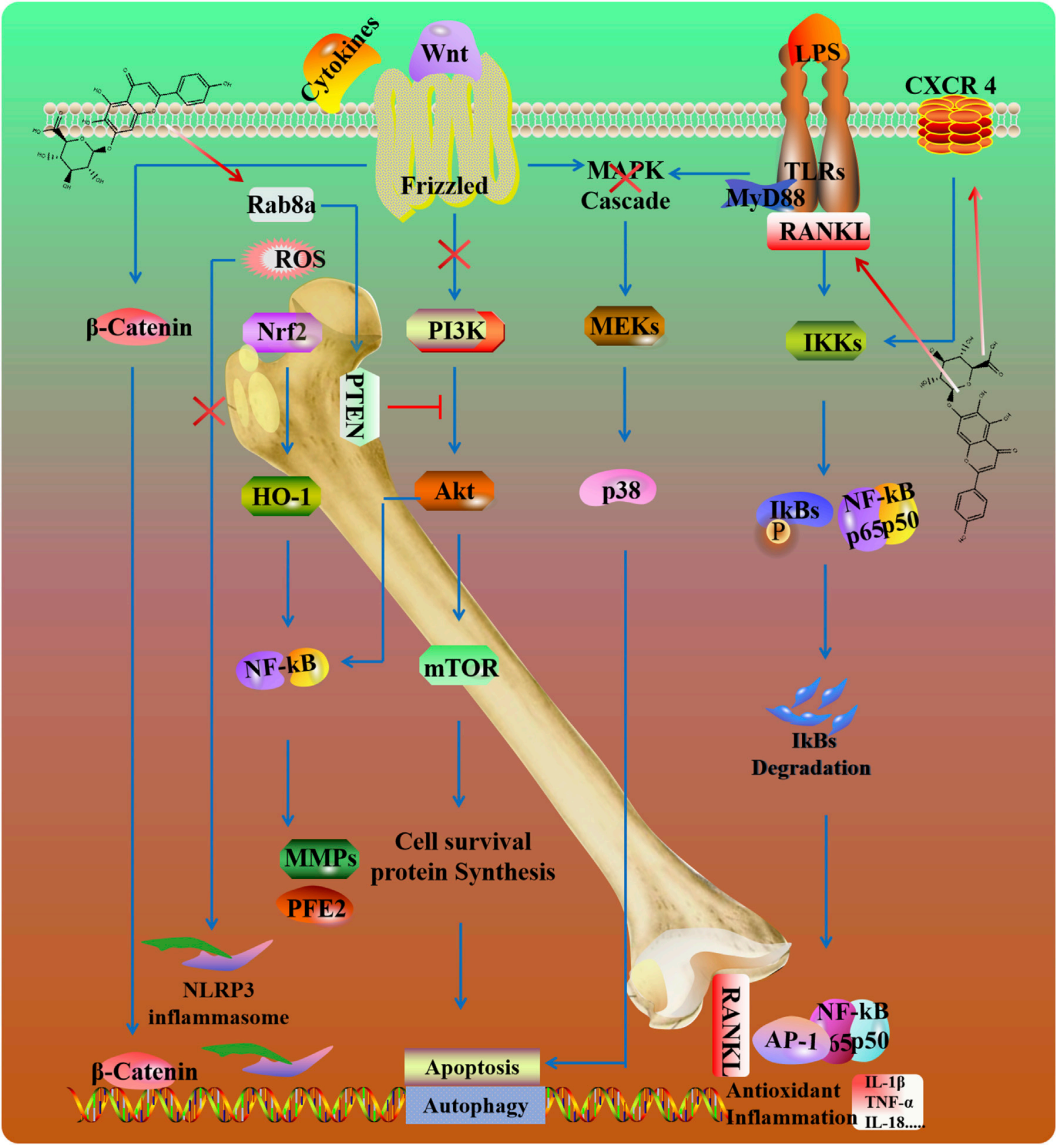
Potential mechanisms and targets of SCU against bone and joint damage.

Osteoarthritis (OA) is a chronic inflammatory joint disease. It is driven by an imbalance between anabolic and catabolic cartilage such as MMP1 and MMP13. SCU inhibited IL-1β-mediated inflammation in chondrocytes, reducing MMP-13, ADAMTS-5, COX-2, and iNOS via NF-κB and Nrf2 pathway (Liu et al., 2020; Luo et al., 2020; Wang et al., 2019). SCU also affects cholesterol metabolism in OA cells by modulating the CH25H/CYP7B1/RAR-related orphan receptor α axis (Ju et al., 2021; Luo et al., 2020; Wang et al., 2019) (Figure 6).

Intervertebral disc degeneration (IVDD) is the most widespread cause of disc herniation. Inflammatory responses, mitochondrial dysfunction, and extracellular matrix degradation are the main etiologies of this disease (Chao-Yang et al., 2021). In a rat needle puncture model, SCU attenuated the inflammatory reaction and retained the production of major intervertebral disc components. Mechanistically, SCU reduced the amount of ROS and alleviated mitochondrial damage by inhibiting NLRP3/NF-κB/MAPK signaling in TNF-α induced human primary nucleus pulposus cells (Wang Z. et al., 2022). Moreover, SCU enhanced autophagy, upregulated the expression of Rab8a and promoted the release of exosomes through the inactivation of PTEN/PI3K/Akt pathway in nucleus pulposus cells (Hu et al., 2022).

Arthritis, characterized by synovitis and hypertrophic synovium (swelling), can be improved by inhibiting inflammation and oxidative stress (Zhang et al., 2017). SCU inhibited RANKL-mediated MAPKs and NF-κB signaling pathways to counter osteoclastogenesis (Zhao et al., 2016). Osteoporosis is characterized by low bone mass and micro-architectural deterioration of bone tissue. C-X-C chemokine receptor type 4 (CXCR4) participates in immune responses and bone remodeling by modulating mesenchymal stem cells and osteoclast precursors' proliferation, maturation, and migration (Liu et al., 2024). SCU improves osteoblast function by increasing the expression of CXCR4 and inhibiting the NF-κB signaling pathway (Wang et al., 2018).

### 2.6 Metabolic diseases

Obesity is characterized by excessive fat deposition. SREBP family, CCAAT-enhancer binding protein (C/EBP) family and other adipogenic transcription factors are involved in the generation of adipogenesis. Emerging evidence reveals that PPARγ acts cooperatively with C/EBPα to mediate adipocyte differentiation (Benchamana et al., 2019). In 3T3-L1 preadipocytes, SCU could attenuate fat cell differentiation by upregulating PPARα and downregulating PPARγ and C/EBP (Li et al., 2009).

Diabetic nephropathy is one of the most frequent and severe complications of diabetes mellitus (DM) and is associated with increased morbidity and mortality in diabetic patients. Oxidative stress, angiotensin II (Ang-II), and inflammatory processes are recently considered to play an important role in the development and progression of DN (Jin et al., 2023). In DN mice, SCU could ameliorates proteinuria, glomerular expansion, mesangial matrix accumulation, renal fibrosis, and podocyte injury by inhibiting TGF-*β* and as well as its interaction with the extracellular signal-regulated kinase (Erk) and Wnt/β-catenin pathways (Huang et al., 2024).

Diabetic retinopathy (DR) is another serious microvascular complication of DM and is the leading cause of visual loss in the elderly, with a prevalence of 34.6% (93 million) in adults aged 40 years and over (Yue et al., 2022). Network pharmacology demonstrated that SCU can effectively protect retina ganglion cells from pyroptosis in DR, and underlying mechanisms are involved in the inhibition of caspase-1, GSDMD, NLRP3, IL-1β and IL-18 (Li N. et al., 2023). The loss of blood-retinal barrier (BRB) integrity leads to ischemic retinal. Claudin-1 and claudin-19 are critical for maintaining BRB integrity. In high glucose and hypoxia-induced human retinal endothelial cells, SCU attenuated HREC proliferation, migration, and tube formation. Meanwhile, SCU decreased neovascularization and resistive index in the retina of diabetic rats. The mechanism of SCU appears to the inhibition the expression of the crosstalk of NLRP3, VEGF, p-ERK, p-FAK, and p-Src and promote the levels of claudin-1, and claudin-19 (Long et al., 2019; Mei et al., 2019; Yang et al., 2024b).

Diabetic cardiomyopathy is a major complication of diabetes and the prominent features are cardiac hypertrophy and fibrosis, which is closely related to autophagy or apoptosis of cardiomyocytes (Lezoualc’h et al., 2023). In the high-fat and high-sugar diet-induced DCM model, SCU alleviated myocardial damage in a dose-dependent manner by promoting the expression of Beclin-1 and LC3-II and decreasing caspase-3, caspase-8, Bax, and other apoptosis-related factors in diabetic cardiomyopathy (Huo et al., 2021; Su et al., 2022). Additionally, SCU reversed high-glucose-induced inflammatory and oxidation stress by inhibiting the NLRP3/NF-κB pathway and enhancing the AKT/Nrf2/HO-1 pathway (Xi et al., 2021; Xu et al., 2021).

Increased advanced glycation end products and free fatty acids lead to diabetic liver injuries (Kumar et al., 2021; Stefan and Cusi, 2022). In a T2DM animal model and homocysteine-induced hepatocyte line LO2, SCU improved liver function, enhanced the clearance of homocysteine and ameliorated hepatic injury. Furthermore, SCU suppressed the secretion of IL-1, IL-6, and TNF-α and reduced hepatocyte apoptosis (Fan et al., 2023; Wang et al., 2020).

### 2.7 Cancer

#### 2.7.1 Lung cancer

Lung cancer is the second most prevalent and the deadliest cancer worldwide. Non-small cell lung cancer (NSCLC) accounts for approximately 85% of lung cancer and the five-year survival rate remains <15% (Miao et al., 2024). Acquired resistance of cisplatin has been a major obstacle for the clinical application. Drug-induced apoptosis and autophagy can sensitize cancer cells to chemotherapy (Lou et al., 2021). SCU enhanced cisplatin-induced autophagy by suppressing the c-met/AKT signaling and apoptosis via enhancing ERK/P53 signaling and further reversing cisplatin resistance (Sun C. Y. et al., 2018). The pro-apoptosis and autophagy efficacy of SCU was also confirmed by another study, which demonstrated that SCU could inhibit the proliferation of A549 cells, induce G0/G1 phase arrest, apoptosis, and autophagy via AKT/mTOR/4EBP1 and ERK1/2/STAT3 pathways (Cao et al., 2019). Moreover, SCU improved the radiosensitivity of non-small cell lung cancer cells to ^125^I seeds by downregulating the AKT/mTOR pathway *in vivo* and vitro in a concentration and time-dependent manner (Cao et al., 2019; Zhang et al., 2021).

#### 2.7.2 Liver cancer

Hepatocellular carcinoma (HCC) is the sixth most common malignancy and the fourth leading cause of cancer-related death worldwide (Brown et al., 2023). JAK/STAT signaling pathway has been documented to arbitrate the transcription pathways of several cytokines in human malignancies, including HCC (Liao et al., 2024). STAT3 is a crucial regulatory molecule in cancer immunity (Kang et al., 2021). Girders of actin filaments (Girdin) are related to poor prognosis of HCC. In HepG2 and MHCC97-H cells, SCU potentially suppresses invasiveness by inhibition of the EMT process, which could be attributed to the downregulation of the JAK2/STAT3/Girdin/Akt pathway (Ke et al., 2017; Liu K. et al., 2019). Immunogenic cell death of cancer cells may induce adaptive immunity against tumors, thereby providing great potential for treating HCC. Li L. et al. (2023) produced an aminoethyl anisamide-targeted polyethylene glycol-modified poly (lactide-co-glycolide) (PLGA-PEG-AEAA) for encapsulating SCU. PLGA-PEG-AEAA.SCU achieved anti-HCC efficacy due to the reversal of immunosuppressive tumor microenvironment, significantly prolonging the survival of orthotopic HCC mice, without inducing toxicity. Recently, Isochlorate dehydrogenase one can limit glycolysis in hepatocellular carcinoma (HCC) cells to activate the tumor immune microenvironment. SCU showed significant anti-hepatoma effects by inhibiting glycolysis, recruiting immune cells into the tumor microenvironment, and blocking PD-L1 expression in transplanted tumor models. In hypoxia induced HepG2 and Huh7 cell, SCU inhibited glycolysis by regulating the IDH1–α-KG–HIF1a signaling axis (Cui et al., 2024).

#### 2.7.3 Colorectal cancer

Colorectal cancer (CRC) is one of the heterogeneous diseases with high morbidity and mortality worldwide. Increasing evidence suggests that Hedgehog signaling plays a pivotal role in the initiation, development, and metastasis of CRC (Geyer and Gerling, 2021). SCU suppressed the proliferation, migration, and colony formation by inhibiting the Hedgehog, Wnt/β-catenin, NF-κB and ephrinb2/VEGF signaling (; Lei et al., 2020; Zeng et al., 2021; Zeng et al., 2022). Regulation of CD4^+^, Foxp3^+^ regulatory T cells (Tregs) is emerging as a potential therapeutic target in CRC. SCU has shown promising effects in reducing the number of tumor-infiltrating TNFR2-positive Tregs and increasing the infiltration of IFN γ-induced CD8^+^ T cells. This shift in the immune environment favors anti-tumor activity and can potentially hinder tumor growth and spread (Chen et al., 2022; Yang et al., 2017). Pyruvate kinase isoenzyme M2 is overexpressed in cancer cells and associated with cancer development. SCU resensitizes oxaliplatin-resistant CRC cells to oxaliplatin treatment through inhibition of PKM2 and reduction of the glycometabolism rate and the production of ATP (Sun et al., 2021).

#### 2.7.4 Gastric cancer and oral squamous cell carcinoma

Gastric cancer is one of the most common malignancies with high mortality, especially in East Asia (Guan et al., 2023). SCU improved enzymatic and non-enzymatic antioxidant profiles and reversed inflammation in N-methyl-N′-nitro-N-nitrosoguanidine induced gastric carcinogenesis model (Sun and Meng, 2022). PTEN is frequently mutated in gastric cancer and is regarded as a tumor suppressor. The mechanistic study supported that SCU silenced PI3K by up-regulating PTEN, thus dampening tumor progression in nude mice (Li et al., 2021). Additionally, SCU suppressed gastric cancer cell proliferation and promoted apoptosis by inhibition of the Wnt/β-catenin pathway in a dose-independent manner (Wang et al., 2023). Oral squamous cell carcinoma (OSCC) is ranked as the sixth most common cancer worldwide, with approximately 900,000 cases and more than 400,000 cases of incidence and mortality rate (Badwelan et al., 2023). SCU inhibited the proliferation and induced apoptosis by reducing the expression of transcription factor AP-1, MMP-2, MMP-9, and integrin αvβ6 in the HSC-4 and SAS human OSCC cells (Li et al., 2013; Li et al., 2010).

### 2.8 Sepsis

Sepsis and septic shock are severe systemic inflammatory responses to infection, that result in physiologic organ system dysfunction (Marshall and Leligdowicz, 2022). The NLRP3/caspase-1/IL-1 axis plays a critical role in the innate immune system and the progression of inflammation. SCU suppressed NLRP3 inflammasome activation in LPS-induced macrophages by enhancing PKA signaling (Liu et al., 2017). Moreover, SCU protects against LPS-provoked AKI by restraining inflammation and oxidative stress. The mechanism appears to regulate Nrf2/PPAR-γ/PGC-1α/NF-κB/TLR4 signaling (Liu et al., 2022; Shahmohammadi et al., 2023).

### 2.9 Toxicity-reducing and efficacy-enhancing

Doxorubicin (DOX), an anthracycline antineoplastic agent, is limited in clinical due to cardiotoxicity. The reduction of oxidative stress, mitochondrial dysfunction, DNA damage, apoptosis, and autophagy has been shown to confer significant protection against doxorubicin (DOX)-induced cardiotoxicity *in vivo* (Kong et al., 2022). SCU attenuation of DOX-induced oxidative stress, DNA damage, mitochondrial dysfunction, apoptosis, and autophagy in H9C2 cells, cardiomyocytes, cardiac fibroblast cells, and human umbilical vein endothelial cells and in rats (Sun et al., 2021; Sun et al., 2023; Tang et al., 2019; Zhou et al., 2022). The pharmacokinetic and tissue distribution study suggested that SCU reduced the concentration of DOX in heart tissues through its antioxidant activity (Sun et al., 2017).

## 3 Conclusion and future perspectives

The pathological development of chronic diseases is intricate due to the multiple signaling pathways involved in these dynamic interactions. The current treatment is still unsatisfactory due to the single or a few molecular targets of the targeted agents. Additionally, these treatments can cause serious side effects, known as “on-target” or “off-target” effects. SCU is a flavonoid that exerts a variety of pharmacological and biological activities, including anti-inflammatory, antioxidant, apoptosis-regulating, and vasodilating properties. However, current research lacks specificity and depth in elucidating how these targets and pathways interconnect within the broader context of each disease. While the referenced review focuses on the anti-inflammatory mechanisms of SCU, our work provides a broader scope, covering its role not only in inflammation but also in cardiovascular diseases, neuroprotection, and ischemia/reperfusion injury. Additionally, we explore novel findings regarding the role of vasodilation and apoptosis regulation, particularly in the context of ischemic stroke and myocardial infarction.

Through the collection of the published articles, most experimental results are preliminary results from cells and rats. The deficiency of positive control leads to a lack of reference for the clinical application of SCU. Despite the promising therapeutic potential of SCU, identifying the key molecular targets of SCU is challenging, and more pharmacological mechanisms of action need to be further explored. Moreover, SCU, when used in combination with other drugs, can enhance therapeutic efficacy, presenting promising potential for future applications.

While the studies provide compelling evidence of SCU’s broad pharmacological effects, including its antioxidant, anti-inflammatory, and cardioprotective properties, several limitations exist in the current body of research. First, many of the studies are preclinical, primarily using cell and animal models, which may not accurately reflect human physiology. The clinical relevance remains uncertain until more human trials are conducted. Second, some studies lack detailed dose-response analyses, which makes it difficult to determine the optimal therapeutic dosage for SCU and raises concerns about potential toxicity or side effects at higher concentrations. Third, poor bioavailability significantly impacts its therapeutic potential. Few studies address effective delivery systems leaving a gap in the practical application of SCU as a therapeutic agent. Future research should focus on advanced drug delivery systems, such as nanoformulations. Co-crystallization and nanoformulation technologies offer an innovative approach to developing combination therapies involving SCU. These technologies improve the therapeutic effectiveness and address key challenges associated with SCU, such as poor stability, low water solubility, limited oral bioavailability, and a short half-life *in vivo*.

While preclinical studies provide compelling evidence of SCU’s therapeutic potential, there is a lack of robust clinical data. Large-scale, randomized controlled trials are needed to validate its efficacy and safety in human populations. Future clinical trials should focus on establishing optimal dosages, long-term safety profiles, and potential drug interactions when used in combination with other therapeutic agents. Although the pharmacological effects of SCU have been well-documented, more detailed mechanistic studies are needed to explore molecular pathways, including its interactions with the NF-κB, PI3K/Akt, and MAPK signaling pathways. This will be helpful in better elucidating its therapeutic mechanisms and identifying potential biomarkers in various chronic diseases. This could help to identify new therapeutic targets and potential biomarkers for various diseases. Additionally, SCU has shown potential in combination with other drugs, which may enhance its therapeutic effects through synergistic mechanisms. Future studies should explore the efficacy of SCU in combination with other standard therapies, focusing on its potential to reduce drug resistance or adverse side effects. Beyond its well-studied effects on cardiovascular, cancer, diabetes, and neurodegenerative diseases, SCU’s anti-inflammatory and antioxidant properties make it a potential candidate for managing other chronic diseases driven by oxidative stress and inflammation. Future research could expand the application of SCU to a wider range of chronic diseases.

In summary, SCU has demonstrated significant potential as a therapeutic agent across a broad spectrum of chronic diseases, including cardiovascular, cerebrovascular, diabetes, organ injury, metabolic disorders, and neurodegenerative disorders. Antioxidant, anti-inflammatory, anti-apoptotic, and vasodilatory activities underscore its therapeutic versatility. SCU demonstrates the ability to modulate multiple signaling pathways in the treatment of chronic diseases, highlighting its promising future applications in preclinical models. Despite the encouraging results in preclinical models, SCU faces several challenges, such as poor bioavailability and limited clinical data, which hinder its broader application in clinical. To fully realize the potential of SCU as a widely applicable drug, more systematic and comprehensive studies are needed to accelerate its development for clinical use.
